# Biophysical and biochemical studies support TP0094 as a phosphotransacetylase in an acetogenic energy-conservation pathway in *Treponema pallidum*

**DOI:** 10.1371/journal.pone.0283952

**Published:** 2023-05-18

**Authors:** Chad A. Brautigam, Ranjit K. Deka, Shih-Chia Tso, Wei Z. Liu, Michael V. Norgard

**Affiliations:** 1 Department of Biophysics, UT Southwestern Medical Center, Dallas, Texas, United States of America; 2 Department of Microbiology, UT Southwestern Medical Center, Dallas, Texas, United States of America; University of Nova Gorica, SLOVENIA

## Abstract

The mechanisms of energy generation and carbon-source utilization in the syphilis spirochete *Treponema pallidum* have remained enigmatic despite complete genomic sequence information. Whereas the bacterium harbors enzymes for glycolysis, the apparatus for more efficient use of glucose catabolites, namely the citric-acid cycle, is apparently not present. Yet, the organism’s energy needs likely exceed the modest output from glycolysis alone. Recently, building on our structure-function studies of *T*. *pallidum* lipoproteins, we proposed a “flavin-centric” metabolic lifestyle for the organism that partially resolves this conundrum. As a part of the hypothesis, we have proposed that *T*. *pallidum* contains an acetogenic energy-conservation pathway that catabolizes D-lactate, yielding acetate, reducing equivalents for the generation and maintenance of chemiosmotic potential, and ATP. We already have confirmed the D-lactate dehydrogenase activity in *T*. *pallidum* necessary for this pathway to operate. In the current study, we focused on another enzyme ostensibly involved in treponemal acetogenesis, phosphotransacetylase (Pta). This enzyme is putatively identified as TP0094 and, in this study, we determined a high-resolution (1.95 Å) X-ray crystal structure of the protein, finding that its fold comports with other known Pta enzymes. Further studies on its solution behavior and enzyme activity confirmed that it has the properties of a Pta. These results are consistent with the proposed acetogenesis pathway in *T*. *pallidum*, and we propose that the protein be referred to henceforth as TpPta.

## Introduction

The obligate human parasite *Treponema pallidum* was identified as the etiologic agent of syphilis over one-hundred years ago [1]. Decades later, the genome of this spirochete was among the first bacterial chromosomes to be fully sequenced and annotated [2]. Despite these many years of study, much of this spirochete’s biology and pathogenicity remain enigmatic, mainly due to the difficulty in culturing the organism *ex vivo*. In particular, the organism’s energy generation and carbon-source utilization have presented a conundrum to researchers: the aforementioned genomic information revealed genes for glycolytic enzymes, but none for those implicated in the citric-acid cycle, oxidative phosphorylation, nor the uptake of carbon sources besides glucose. It thus appeared that *T*. *pallidum*’s sole source of energy was glucose, and yet this resource is utilized inefficiently. This supposition conflicted with the organism’s robust tissue invasiveness and extensive motility [3, 4].

Over the past two decades, we have engaged in a comprehensive structural and functional survey of the lipoprotein (LP) complement of *T*. *pallidum*. LPs are proteins that exist in the organism’s periplasm and are anchored to the cytoplasmic or outer membrane via lipid anchors attached to their respectively processed amino-termini. Among the many discoveries engendered by this work, several have been germane to energy generation in *T*. *pallidum*. First, the spirochete, which is auxotrophic for flavin-based nucleotides, employs an unusual means of importing riboflavin: an ABC-transporter system with a periplasmic ligand-binding protein [5]. Most bacteria use ECF transport systems for this purpose [6–8]. Next, a periplasmic enzyme called TpFtp has the dual function of catalyzing the pyrophosphorolysis of FAD and of transferring FMN moieties to periplasmic proteins (i.e. “flavinylation”) [9, 10]. Finally, closer scrutiny of the *T*. *pallidum* genome revealed a particular focus on flavin-containing biomolecules (collectively “flavins” herein). To wit, the bacterium appears to utilize flavodoxins rather than ferredoxins, it has a flavin-salvage pathway, and it contains a putative RNF complex that can use flavin reducing equivalents to drive the formation and maintenance of a chemiosmotic gradient between the periplasm and the cytoplasm [3].

**Fig 1:**
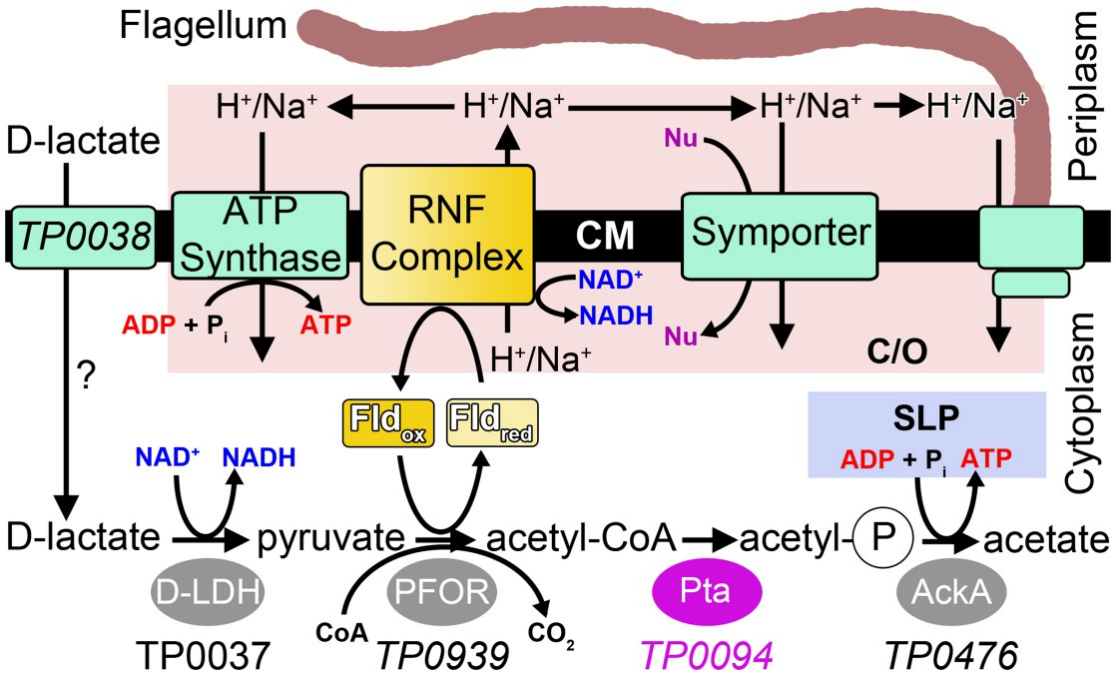
A putative acetogenic pathway in *T*. *pallidum*. Enzymes in the pathway are shown near the bottom of the figure as ovals; the focus of this work is highlighted in purple. Flavoproteins are shown in yellow shades, and transmembrane proteins and complexes are depicted as cyan rectangles except for the RNF complex, which, as a potential flavoprotein, is shaded yellow. Proteins that participated in the formation and utilization of the chemiosmotic gradient are highlighted in a pink box labeled “C/O.” Substrate-level phosphorylation by the putative AckA is highlighted in a light-blue box labeled “SLP.” The question mark indicates that the role of TP0038 in the import of D-lactate is hypothesized but not established. “Nu” stands for “nutrient.” This figure was adapted (changes were made) from Fig 1 in [11]. The license under which this was done is at https://creativecommons.org/licenses/by/4.0/.

When considered together, these discoveries and observations have led us to propose a “flavin-centric” metabolic lifestyle for *T*. *pallidum* [3] that incorporates a reliance on flavins and helps to resolve the energy-generation puzzle posed above. A key aspect of this hypothesis is the existence of an acetogenic biochemical pathway in this bacterium (Fig 1). This pathway, in which the alternative carbon source D-lactate is eventually catabolized to acetate, provides flavin-based reducing equivalents for the function of the RNF complex and yields an ATP molecule through substrate-level phosphorylation. Although all of the enzymes necessary for this acetogenic pathway are apparently present in the genome of *T*. *pallidum*, we have confirmed the activity of only one of them: the D-lactate dehydrogenase encoded by gene *tp0037* (TpDLD; 1.1.1.28; UniProt Acc. # O83080) [11].

To continue the validation of this pathway, thus creating a more comprehensive view of *T*. *pallidum* metabolism, we have characterized the protein TP0094 (UniProt Acc. #O83132), the putative phosphotransactetylase (Pta; EC 2.3.1.8) catalyzing the penultimate step in the acetogenic pathway, i.e., displacing the CoA moiety of acetyl-CoA with a phosphate, yielding acetyl phosphate (Fig 1). Extensive biophysical study of the protein, including X-ray crystallography and solution biophysics, established its tertiary and quaternary structures and allowed for the comparison of these aspects to other known Pta enzymes. We also employed *in vitro* biochemical assays to verify the activity of the protein.

## Materials and methods

### Protein expression and purification

To produce a recombinant derivative of TP0094 in *Escherichia coli*, the DNA fragment encoding all amino-acid residues of TP0094 was PCR amplified from the *T*. *pallidum* genomic DNA (as sourced in [12]) by the polymerase incomplete primer extension (PIPE) cloning method using ends-specific primers (PIPE insert; the primers are specified in S1 Table). The expression vector, pSpeedET (DNASU, AZ), which encodes an N-terminal expression and purification hexa-histidine tag (MGSDKIHHHHHHENLYFQG), was PCR amplified with PIPE-vector primers. The PIPE-insert and PIPE-vector were mixed to anneal the amplified DNA fragments together [13]. *E*. *coli* HK100 competent cells were transformed with the mixtures (PIPE-vector and insert) and selected for kanamycin resistance on LB agar plates. Cloning junctions/fragments were verified by DNA sequencing. The plasmid was then co-transformed with pGroESL (Takara) into *E*. *coli* BL21 AI (Invitrogen) cells for soluble protein expression. *E*. *coli* BL21 AI cells were grown at 37 °C in LB medium containing 40 μg/mL of kanamycin and 30 μg/mL of chloramphenicol until the cell density reached an A_600_ of ~0.6. The cells were then induced for ~20 h with 0.2% (w/v) L-arabinose at 16 °C and harvested; cell pellets were stored at −80 °C. The procedures for expression and purification of the recombinant proteins were essentially as previously described [10, 14]. Purified proteins were stored in Working Buffer (20 mM HEPES, 0.1 M NaCl, pH 7.5, 2 mM n-Octyl-β-D-glucopyranoside) at 4 °C until used.

### Site-directed mutagenesis and protein concentration determination

For the construction of structure-guided TP0094 variants (S314A, R315A and D321A), each mutation was individually introduced into the plasmid carrying the wild-type *tp0094* sequence using the QuikChange site-directed mutagenesis kit (Agilent Technologies). DNA oligonucleotides used for mutagenesis are elaborated in S1 Table. The mutation was confirmed by DNA sequencing. The mutant proteins were expressed and purified as described above. Protein concentrations were determined in Working Buffer using UV absorption at 280 nm. Extinction coefficients were calculated from the protein sequences using the ProtParam tool of ExPASy server (www.expasy.org).

### Pta assays

The phosphotransacetylase (Pta) activity of TP0094 was measured by monitoring the CoA release from acetyl-CoA in the presence of inorganic phosphate, taking advantage of the fact that Ellman’s reagent (5,5’-dithio-bis(2-nitrobenzoic acid), DTNB) efficiently reacts with CoA to form the yellow-colored 2-nitro-5-thiobenzoate anion (TNB) [15]. The steady-state kinetic assays were conducted in an Agilent 8453 diode-array UV Vis spectrophotometer by acquiring the time trace of the absorption at 412 nm; the temperature-controlled cuvette holder was connected to a circulating water bath set to 37 °C. The assay mixture contained 50 mM Tris-HCl, pH 8.0, 20 mM KCl, 0.1 mM DTNB, 0.4 mM Acetyl-CoA, and 75 mM KH_2_PO_4_. For a typical 1 mL assay, the enzymatic reaction was initiated by mixing 10 μL of TP0094 solution (0.02–12 mg/mL; 0.5–300 μM on a monomer basis) into the pre-incubated assay mixture; the time trace in the first 5 s for WT TP0094 or the first 3 sec for all the mutant TP0094 proteins was fitted to a straight line and the slope was taken as the initial rate (*V*_0_). The initial rates were maintained within 0.02–0.08 ΔA_412_/s by varying the TP0094 concentration in any given assay. The molar extinction coefficient value 13,600 M^−1^cm^−1^ at 412 nm for TNB was used to convert the initial rate to the reported activity units (μmol/min).

### Analytical ultracentrifugation

Analytical ultracentrifugation in the sedimentation velocity (SV) mode was carried out in a Beckman Optima XL-I centrifuge (Beckman-Coulter, Inc.). Centrifugation cells were assembled by sandwiching charcoal-filled Epon 1.2-cm centerpieces between sapphire windows in aluminum cell housings. After proper torquing, the samples and Working Buffer were introduced into the sample and reference sectors, respectively, using the external fill ports. The volumes were approximately 400 μL. The filled cells were sealed and placed in an An50-Ti rotor, which was subsequently positioned in the centrifuge. After activating the vacuum pump, the samples were equilibrated at the experimental temperature (20 °C) for about 2 h prior to the commencement of centrifugation at 50,000 rpm. The on-board Rayleigh interference optical system was used to acquire the concentration profiles. After time-stamp correction [16] using REDATE (www.utsouthwestern.edu/research/core-facilities/mbr/software), the data were analyzed using the *c*(*s*) methodology in SEDFIT [17]. An *s*-resolution of 100 was used, with *s*_min_ and *s*_max_ set to 0 and 15 S, respectively. Both radially and time-independent noise were accounted for in the data [18]. A regularization level of 0.683 was used. For wild-type Tp0094, three concentrations were used: 10 μM, 31 μM, and 100 μM. The resulting *c*(*s*) distributions were integrated and illustrated using GUSSI [19].

### Mass photometry

Mass photometry experiments were conducted using a Two^MP^ instrument (Refeyn Ltd). An 8-well silicone gasket was adhered to a glass cover slip (ThorLabs), which was subsequently positioned on the instrument’s mobile stage. Typically, 16.2 μL of PBS was placed into a well, and focusing procedures were undertaken on the water-glass interface. Then, 1.8 μL of the sample, at an approximate concentration of 100 nM (diluted in PBS), was added, followed by mixing by pipetting. A 60-s interferometric movie of a 46 μm^2^ area of the cover slip was acquired. The movie was analyzed in Discover^MP^. The software converted the interferometric movie to a ratiometric one, then it identified, classified, and tabulated all contrast events, presenting them as histograms. Bovine serum albumen (BSA; Sigma-Millipore) at a concentration of 2 μg/mL in PBS was used as the calibration standard, providing four calibration points (BSA monomer through tetramer, i.e., 66–264 kDa). After the contrasts were converted to masses via this standard, the data were exported, and gaussian curves were fitted to histogram peaks using a custom Python script, with the mean of the peak serving as the contrast/molecular-mass estimate and the sigma serving as an estimate of the error in the mass determination. The molecular mass reported in the main text is the mean of four replicates weighted by the respective sigmas.

### Circular dichroism spectroscopy

Circular dichroism (CD) spectroscopy was conducted in a Jasco J815 spectrometer with Peltier temperature control. CD data were monitored at 222 nm, and the temperature was ramped from 25–70 °C at a rate of 1 °C/min. The data were fitted with the following formula:

$$CD(T)=\frac{\left(b_{1}+m_{1}T\right)+\left(b_{2}+m_{2}T\right)e^{-\Delta H\left(1-T/T_{m}\right)/RT}}{1+e^{-\Delta H\left(1-T/T_{m}\right)/RT}},$$ (1)

where Δ*H* is the molar enthalpy of protein folding, *R* is the gas constant, *T* is the temperature in kelvins, *T*_*m*_ is the “melting temperature,” and *b* and *m* are parameters describing the intercepts and slopes, respectively, of the pre-transition region (subscript 1) and the post-transition region (subscript 2). Because a reverse-ramp of the temperature was not performed, Δ*H* is not reported, and *T*_*m*_ is reported as an apparent value, *T*_*m*,app_. Confidence intervals on *T*_*m*_ were calculated using the error-surface-projection method built into the Python module lmfit.

### Structure determination and refinement

Diffraction-quality crystals of TP0094 (with the affinity tag intact) were grown using the sitting-drop vapor-diffusion method. A solution of TP0094 at 12.5 mg/mL (200 nL in Working Buffer) was mixed with an equal volume of the well solution (0.2 M ammonium citrate dibasic, 20% (*w*/v) PEG 3350) and was equilibrated against the well solution for 7 days. The crystals were transferred to the stabilization buffer containing reservoir solution supplemented with 5% (*v*/*v*) ethylene glycol (EG). The crystals were then serially transferred to solutions having higher concentrations of EG in increments of 10%, ending with 35% EG. After about 1 min in this final solution, the crystals were flash-cooled in liquid nitrogen and were stored under liquid nitrogen until used.

Diffraction data from Tp0094 crystals were acquired at beamline 19-ID of the Structural Biology Center at Argonne National Laboratories. The crystals had the symmetry of space group P3_2_21 and diffracted to *d*_min_ spacing of 1.95 Å (Table 1). The data were integrated and scaled using the HKL2000 package [20]. Negative intensities were treated and the data were put on an absolute scale using the method of French & Wilson [21]. Molecular replacement (MR) was accomplished using the Phenix implementation of Phaser [22]. The model used for MR was generated using the Colab-notebook implementation [23] of AlphaFold2 [24]; the native sequence of TP0094 was supplied to the algorithm, and amber-based “relaxation” of the resultant structures was applied. The highest-rated model was chosen for the MR procedure; it was modified by setting all *B*-factors to approximately 25 Å^2^. Phaser located two monomers of the model in the asymmetric unit. The positioned model was subjected to simulated annealing, positional, and individual *B*-factor refinement using Phenix [25]. Manual adjustments to the model were made as necessary in Coot [26]. The final model had excellent refinement (*R*_work_ = 0.174; *R*_free_ = 0.212) and geometric (MolProbity score = 1.4) statistics (see Table 1 for comprehensive statistics).

**Table 1:** X-ray diffraction data and refinement statistics.

**PDB Accession No.**	8FIR
**Data Collection**	
Space Group	P3_2_21
Unit Cell Dimensions (Å)	
a	66.2
b	66.2
c	337.6
α (°)	90
β (°)	90
γ (°)	120
Resolution (Å)	36.9–1.95 (1.98–1.95)^a^
Completeness (%)	98.4 (99.0)
Multiplicity	3.6 (3.0)
Unique Reflections	63,299 (3,101)
*R*_*merge*_ ^b^	0.080 (0.613)
<*I*>/*σ*_*I*_	14.6 (2.1)
Wilson *B* (Å^2^)	24.8
**Refinement**	
Resolution (Å)	36.9–1.95
No. Residues	660
Missing Residues	A334-A336; B334-B336
No. Non-Protein, Non-Solvent Atoms^c^	21
No. Solvent Atoms	125
Maximum-Likelihood	
Coordinate Error (Å)	0.16
**Average *B*-factors**	
Protein (Å^2^)	33.3
Solvent (Å^2^)	38.3
***R*-values**	
*R*_*work*_ ^d^	0.180
*R*_*free*_ ^e^	0.218
**Ramachandran Statistics**	
Outliers (%)	0.0
Most Favored Region (%)	97.7
**r.m.s. deviations**	
Bonds (Å)	0.011
Angles (°)	1.1

^a^Numbers in the parentheses are reported for the highest-resolution shell of reflections.

^b^ $R_{merge}=\sum_{hkl}\sum_{i}\left|I_{h,i}-\langle I_{h}\rangle \right|/\sum_{hkl}\sum_{i}I_{h,i}$ where the outer sum (*hkl*) is over the unique reflections and the inner sum (i) is over the set of independent observations of each unique reflection.

^c^Does not include riding hydrogen atoms.

^d^$R_{work}=\sum_{hkl}\left|\left|F_{o}\right|-\left|F_{c}\right|\right|/\sum_{hkl}\left|F_{o}\right|$, where *F*_*o*_ and *F*_*c*_ are observed and calculated structure factor amplitudes, respectively.

^e^*R*_*free*_ is calculated using the same formula as *R*_*work*_, but the set *hkl* is a randomly selected subset (5%) of the total structure factors that are never used in refinement.

## Results & discussion

### The crystal structure of tp0094

**Fig 2:**
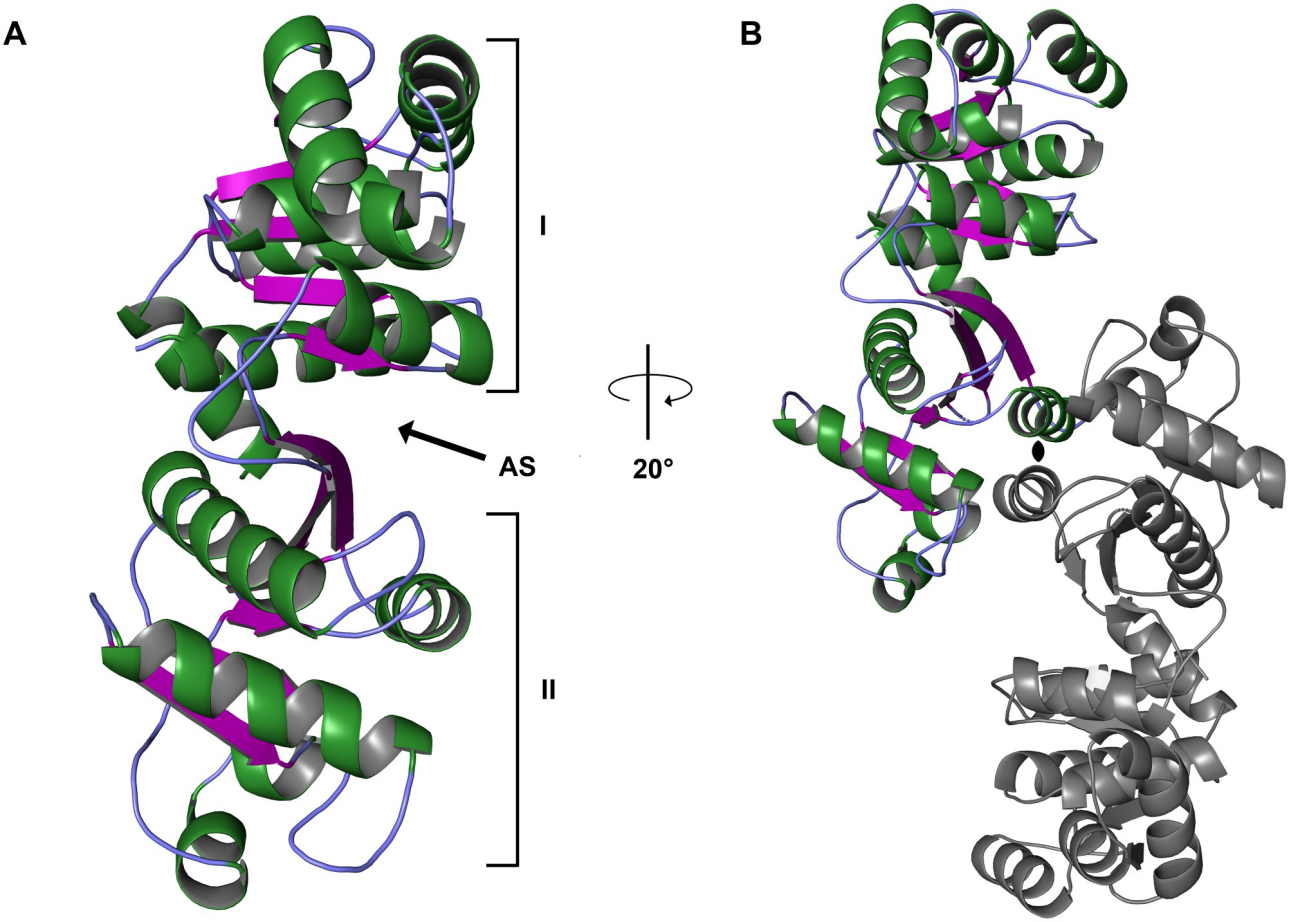
The structure of TP0094. (A) Ribbons-type representation of the structure. Helices are colored green, β-strands purple, and regions of irregular secondary structure light blue. The structure is oriented to emphasize the cleft between the domains that houses the proposed active site (“AS”). Only the “A” monomer of the two protein copies in the asymmetric unit is shown. (B) The dimer of TP0094. The “A” monomer is shown in color, and the “B” monomer in gray. The view has been rotated 20° to emphasize the symmetry between the two polypeptides. The black symbol in the middle marks the approximate position of the pseudo-twofold axis of symmetry.

The crystal structure of TP0094 was determined and refined at a resolution of 1.95 Å (Table 1, Fig 2). There were two molecules in the asymmetric unit, and nearly all residues present in the protein construct could be modeled (see Table 1). The structure is divided into two domains, called “Domain I” and “Domain II” according to established nomenclature for Pta enzymes [27, 28]. Domain I, (amino-acids 1–141 and 309–334), which contains both the amino- and carboxyl-termini, has a central, 5-stranded parallel β-sheet that is flanked by several helical elements. Domain II (amino-acids 150–305) also features a mostly parallel central β-sheet, but the penultimate of its six strands is antiparallel. The sheet is surrounded by helices, like that of Domain I. As noted before [28], the two β-sheets are arranged side-by-side and give the appearance of a single 11-stranded β-sheet, but there are no direct hydrogen bonds between the sheets of Domain I and Domain II, and thus they must be considered as discontinuous.

Between the two domains is a cleft that is implicated in catalysis by other Ptas [29, 30]. Although several water molecules are observed in this cleft, there was no evidence of substrates or products bound therein. The cleft is replete with side chains of polar amino-acids, and thus the potential for binding polar ligands is clear.

Two monomers of the TP0094 polypeptide are present in the asymmetric unit of the crystal (Fig 2B), comprising the A and B monomers of the protein. Monomer-monomer contacts are made solely through the respective Domains I. Monomer A and Monomer B have essentially the same conformation: superposing the 324 common C_α_ atoms of the monomers results in a root-mean-square deviation (r.m.s.d.) of 0.5 Å. The two monomers are intimately associated, burying 2,640 Å^2^ of surface area, and estimates of the energetics of this dimer using PISA [31] indicate a strong likelihood that the dimer is stable in solution (i.e. Δ*G*^int^ = −22.6 kcal/mol).

### Comparisons to Pta enzymes

**Fig 3:**
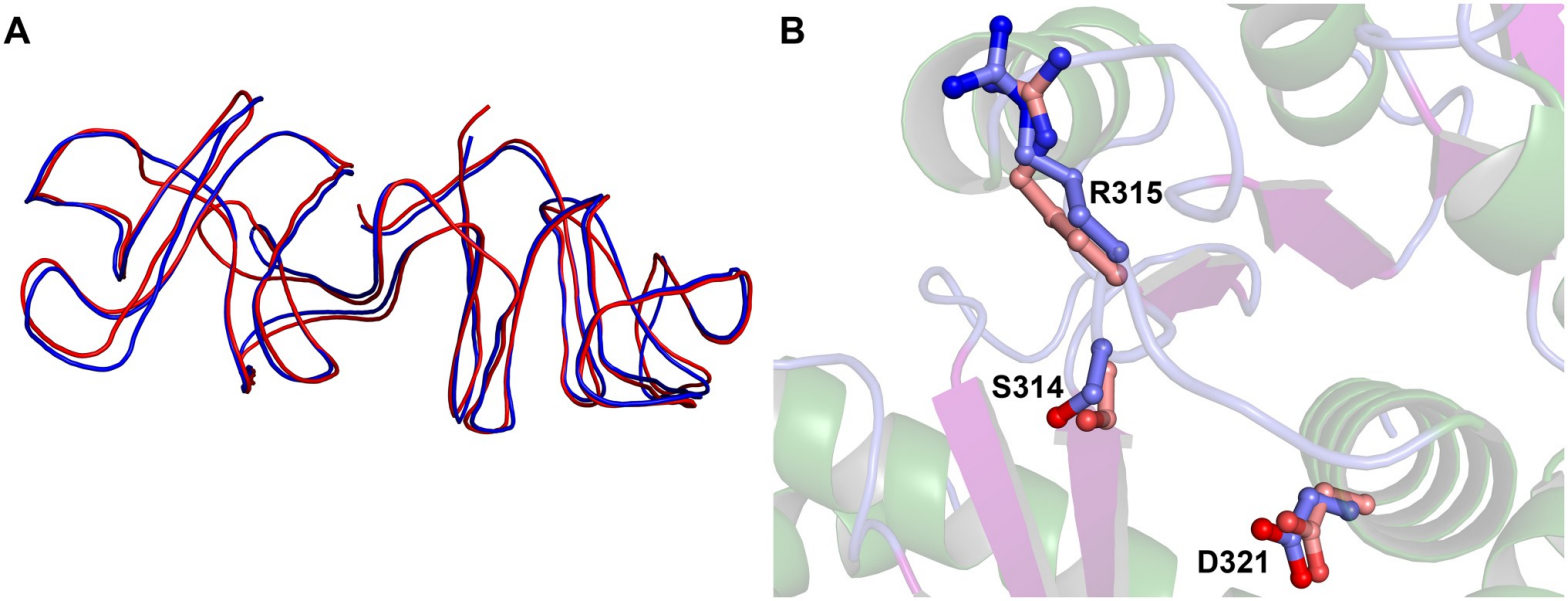
Comparisons of TP0094 and MtPta. (A) Superposition of the monomers. Monomer A of TP0094 (blue) and monomer C of MtPta (PDB Acc. #1QZT [28]; red) are superposed. The lines are smoothed traces through the main-chain atoms of the respective models. (B) Active-site residues. The three residues from TP0094 mentioned in the text (light-blue carbon atoms) are shown in ball-and-stick format, as are their counterparts in MtPta (pink carbon atoms). Secondary-structure elements from the TP0094 structure are shown semi-transparently for clarity.

When structural databases are searched with the TP0094 structure using heuristic [32] and secondary-structure-matching (SSM; [33]) approaches, the highest similarities found are to Pta enzymes. In particular, the structure of the Pta from *Methanosarcina thermophila* (MtPta) is close to that of TP0094, with the SSM algorithm matching 320 C_α_ atoms of TP0094’s Monomer A to the C chain of MtPta (PDB Acc. #1QZT) [28] with an r.m.s.d. of 1.14 Å over 326 aligned C_α_ atoms (Fig 3, S2 Table). Other prominent matches to deposited structures include Ptas from *Porphyromonas gingivalis* (PgPta; PDB Acc. #6IOX; r.m.s.d. of 1.35 Å over 325 aligned C_α_’s; [30]) and *Bacillus subtilis* (PDB Acc. #1TD9; r.m.s.d. of 1.71Å over 319 C_α_’s) [34]. A more comprehensive listing of these results is found in S2 & S3 Tables.

The close structural match of TP0094 to MtPta and the extensive research performed on the latter suggest that this comparison bears particular scrutiny. MtPta has exhibited three distinct conformations in its crystal structures; indeed, all three are present in crystals of the apo-form of the enzyme [28], which feature two dimers in the asymmetric unit. Although MtPta has the same dimeric arrangement in the crystalline state as TP0094, the individual monomers of the *M*. *thermophila* protein can assume divergent conformations. For example, the A and B monomers of the MtPta apo structure differ by a 20° rigid-body rotation of Domain II relative to Domain I, thus sampling “open” and “closed” conformations, respectively. Moreover, the C and D monomers adopt an “intermediate” conformation in which Domain II is rotated only by about 5–7° relative to the open form. It is this intermediate conformation that most resembles the conformation displayed by both monomers of TP0094 (Figs 2 & 3; S1 & S2 Tables). The intermediate conformation of MtPta has only been observed in apo structures of MtPta [28], and the substrate-bound structure of PgPta was in the open conformation [30]. Thus, the conformations of Pta may be important for the enzyme’s catalytic cycle, and the intermediate conformation of TP0094 comports with its lack of substrates or products in its putative active site.

**Fig 4:**
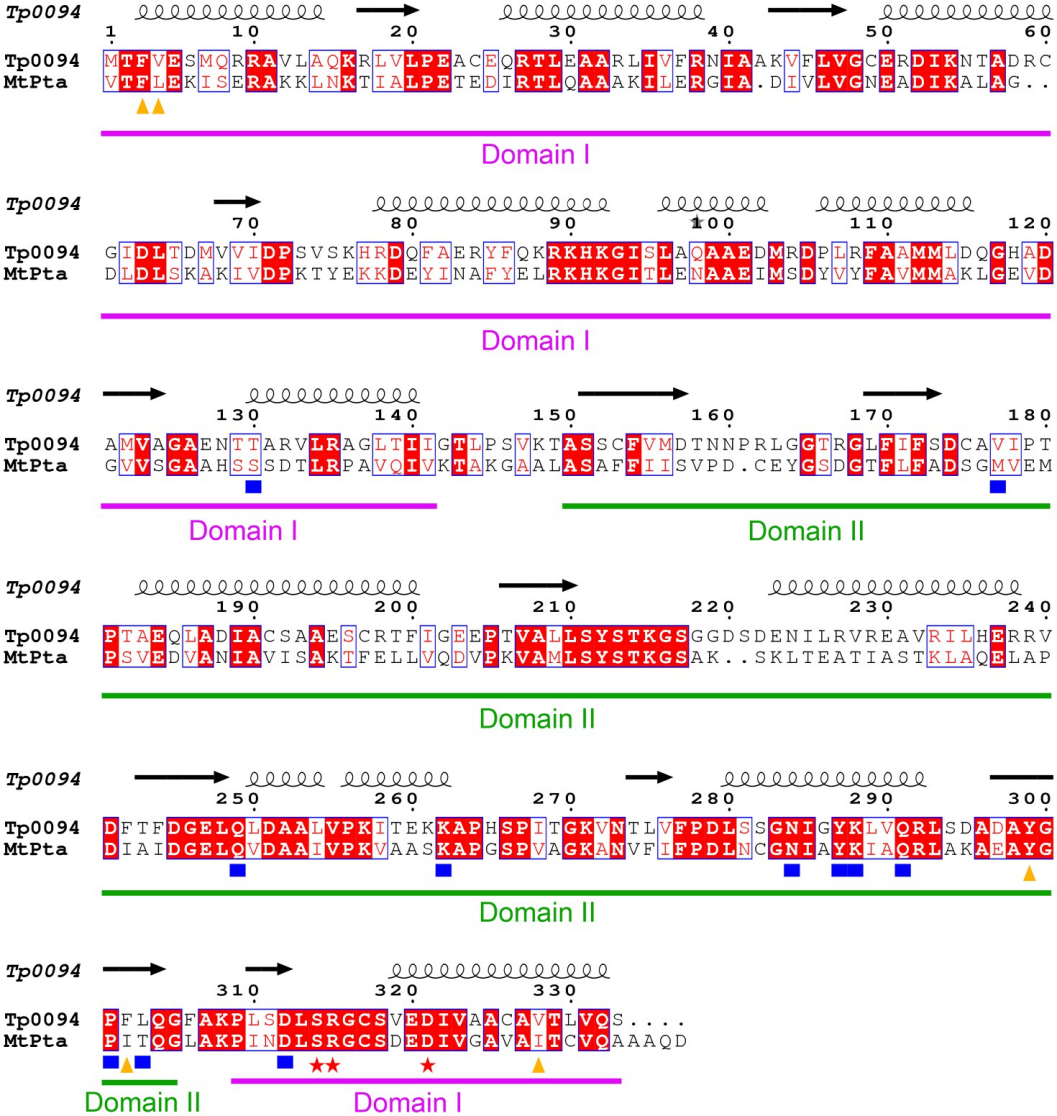
Structure-based alignment of TP0094 and MtPta. The sequences of TP0094 and MtPta, as found in their respective PDB files (Acc. numbers 8FIR and 1QZT, chain C, respectively) are shown as aligned by PROMALS3D [35]. The numbering is from TP0094, and secondary structural elements, as defined by those found in the TP0094 structure, are shown above the alignment; α-helices are coils and β-strands are arrows. Residues highlighted in red are identical, and those outlined in blue have chemical similarity. Orange triangles mark residues that putatively contact acetyl phosphate, blue squares mark putative CoA-contacts, and red stars mark the three putatively catalytic residues. The boundaries of Domains I (purple) and II (green) are shown with lines. The figure was generated using ESPript 3.0 (https://espript.ibcp.fr [36]).

Structural alignment of the MtPta intermediate conformation and Tp0094 showed that most of the substrate-contacting and purportedly catalytic residues are conserved (Fig 4 & Table 2). Indeed, 13 of the 19 identified residues are identical between the two proteins, and this includes three residues that are likely to be involved in catalysis: S314, R315, and D321 (TP0094 numbering). Indeed, the configuration of the active-site residues is very similar when comparing the MtPta and TP0094 structures (Fig 3B). Many of the non-identities revealed by this comparison involve chemically similar replacements (e.g., I297 in MtPta is equivalent to F302 in TP0094). One amino acid in MtPta having a role in CoA binding is not conserved in TP0094; in MtPta, this residue is T298, and its structural counterpart in TP0094 is L303. In CoA-bound MtPta structures, the hydroxyl moiety from the side chain of T298 forms hydrogen bonds with the adenine ring of CoA. Although there is no direct evidence that these hydrogen bonds are essential for CoA binding to MtPta, the branched aliphatic side chain of L303 is clearly unable to accomplish this task and may instead aid binding via van der Waals interactions. It should be noted that, in this analysis, we considered as CoA-binding residues those that putatively contact a CoA that becomes bound in the interdomain cleft; a second CoA-binding site is present in MtPta at the entrance to the cleft [29, 30], but its role in catalysis is unknown.

**Table 2:** Residue comparison between MtPta and TP0094.

Putative Role	MtPta Residue	TP0094 Residue
acetyl phosphate binding	F4	F3
	L5	V4
	Y294	Y299
	I297	F302
	I323	V328
acetyl-CoA binding	S128	T130
	M174	V177
	Q244	Q249
	K257	K262
	N279	N284
	Y282	Y287
	K283	K288
	Q286	Q291
	P296	P301
	T298	L303
	D307	D312
catalytic	S309	S314
	R310	R315
	D316	D321

### Solution oligomeric state of TP0094

Although there is evidence that the solution oligomeric state of Ptas is constitutively dimeric, these conclusions were often based on size-exclusion chromatography [28, 30, 37], which can lead to inaccuracies due to shape aberrations or protein-matrix interactions. Two studies reported the results of sedimentation equilibrium experiments, but only a single speed and concentration of the protein were considered [29, 37]. Here, to more rigorously determine the oligomeric state of TP0094, we used two methods that are not susceptible to potential experimental drawbacks: analytical ultracentrifugation in the sedimentation velocity mode (SV) and mass photometry (MP).

**Fig 5:**
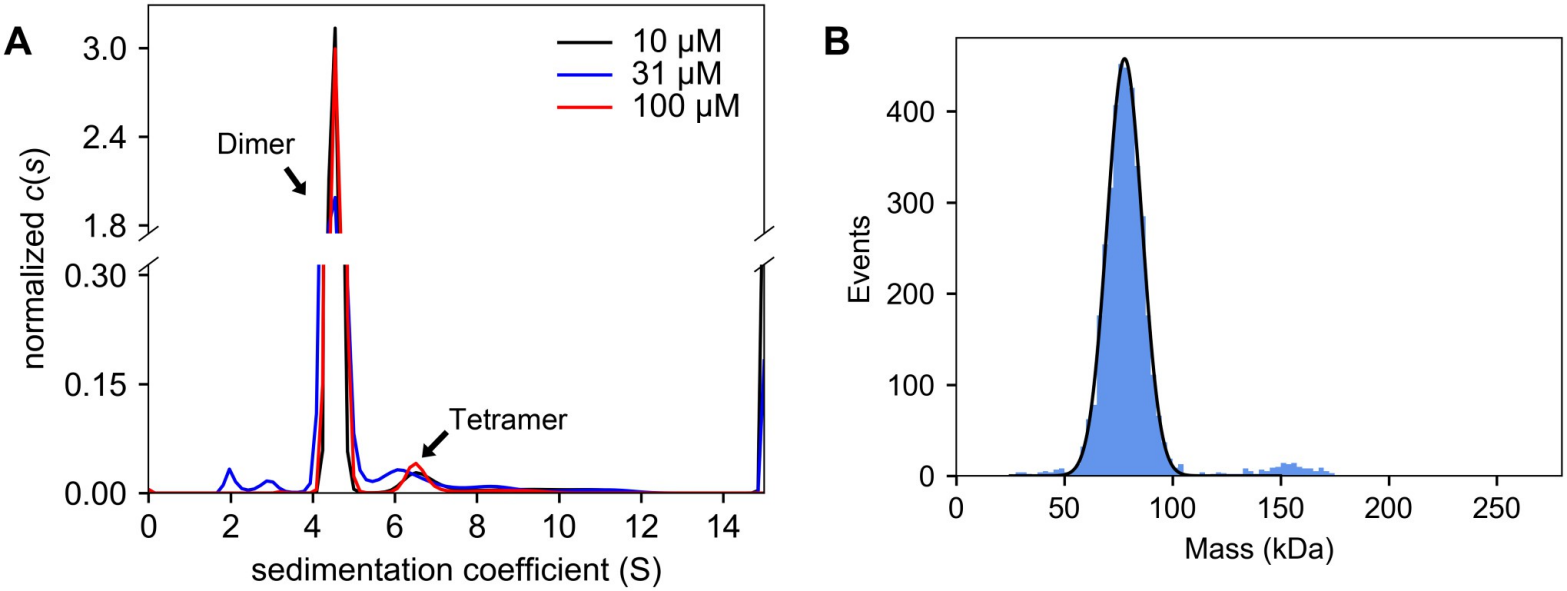
Solution-biophysics characterization of TP0094. (A) SV studies. Three *c*(*s*) distributions are shown, resulting from data at three different concentrations of the enzyme. Correspondence of the distribution colors with the concentrations is shown in the inset legend. (B) MP studies. The histogram from the first replicate of MP data is shown (blue) with a gaussian curve fitted to the main peak (black).

SV experiments demonstrated that there was no discernible trend in the sedimentation coefficient (*s*-value; ca. 4.5 S for the major peaks) of TP0094 as a function of concentration (Fig 5A). The average molar mass derived from these three experiments, 76 ± 2 kg/mol (mean ± SDOM), implied that the protein is dimeric under the solution conditions studied (the formula weight of a dimer is 77.69 kg/mol). When scrutinized carefully, there were secondary, minor peaks present in all the *c*(*s*) distributions we examined (Fig 5A). The *s*-values of these peak (6.3–6.6 S) were consistent with a dimer of the TP0094 dimer, i.e., a tetramer. However, the vast majority of sedimenting material was dimeric (we estimate the mass purity of the dimer to range from 95–97%).

MP was also used to examine the oligomeric state of TP0094, albeit at much lower concentrations (ca. 10 nM; Fig 5B). Again, the data were dominated by a single species, and analysis of this peak in the mass histogram resulted in a molecular mass consistent with a dimer of TP0094 (77.96 ± 0.15 kDa; weighted mean ± weighted SDOM). Another result of these experiments was that, despite the low concentrations employed, there was an apparent lack of dissociation of the dimer into monomers during the time course of the experiment, suggesting a very favorable dimerization constant and/or a very slow dissociation rate constant. As with the SV experiments, a tetramer of TP0094 was evident (Fig 5B). We estimated the molar fraction of this species to be 3.0% ± 0.3%.

The functional relevance of the tetrameric form of TP0094 that was consistently detected in our solution biophysics studies (Fig 5) is questionable. No stable tetrameric form was identified by the computer algorithm employed by PISA [31], which was designed to find protein assemblies from crystal structures. Manual examination of crystal contacts also failed to find a tenable tetrameric form. No tetrameric assembly of Ptas is available in the PDB, although the Pta domains from the hybrid malic enzymes assemble into a hexamer (i.e., a trimer of dimers [38]). TP0094 has three surface-exposed cysteine residues (C24, C49, C60), and thus there is the possibility that the observed tetramers resulted from the disulfide linkage of two dimers. Given the lack of structural or functional evidence or precedent for tetramer formation, we concluded that they are likely artifactual in the case of TP0094.

### The enzymatic activity of TP0094

To assess the enzymatic activity of TP0094, the ability of the protein to release coenzyme A (CoA) in the presence of acetyl-CoA and inorganic phosphate was assayed (Fig 1). We found that TP0094 was able to efficiently catalyze this release, and thus, presumably, the formation of acetyl phosphate. The normalized initial velocity of acetyl-CoA formation was 150 U/mg (Table 3).

**Table 3:** Enzyme activities of wild-type and mutant TP0094 constructs.

Protein	n	Activity (U^a^/mg)	% of WT activity
wild-type	5	150 ± 6^b^	100
S314A	4	0.44 ± 0.05	0.30
R315A	3	0.11 ± 0.00	0.07
D321A	4	21.4 ± 0.9	14.3

^a^One unit (U) is defined as 1 μmol/min of CoA formation.

^b^Activities are presented as the mean ± standard error

### Structural and enzymatic examination of the active site

**Fig 6:**
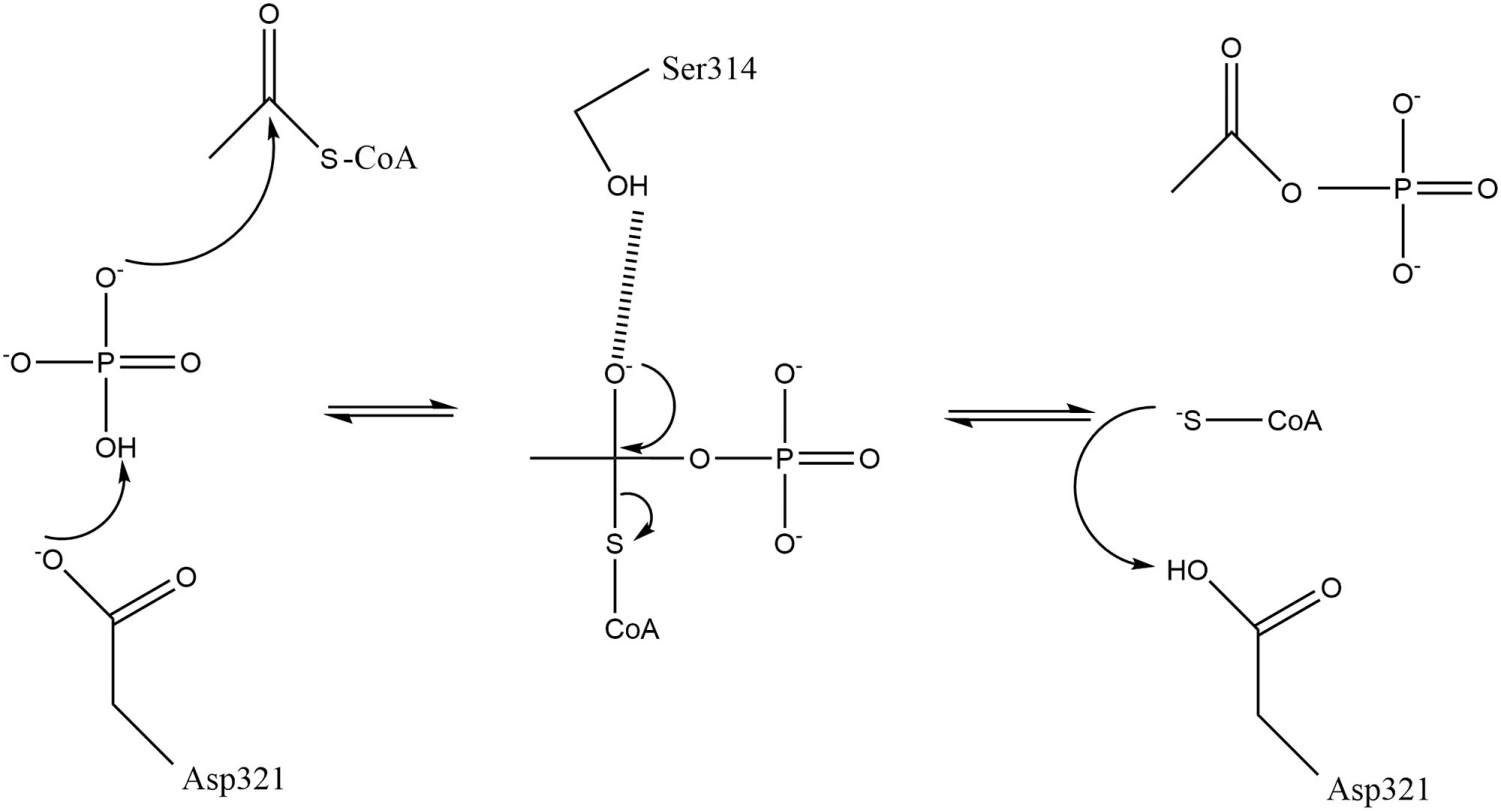
The putative mechanism for the TP0094-catalyzed formation of acetyl phosphate from acetyl-CoA and inorganic phosphate. Residue numbering is that of TP0094. The dashed line between the hydroxyl moiety of Ser314 and the tetrahedral intermediate is a proposed hydrogen bond. Adapted with permission from Fig 7B of [29].

A significant amount of structure-based enzymology has been conducted using MtPta [28, 29], and thus this enzyme was used as the basis of comparisons for the evaluation of the active site of Tp0094. Previous studies on MtPta had implicated the cleft between Domains I and II in catalysis, as it is the site of CoA binding [29]. Indeed, a series of crystal structures in the presence and absence of substrates and products, in addition to enzymatic studies, provided sufficient data for a mechanism of enzyme action to be proposed [29]. Although this mechanism was formulated for the reverse reaction that we consider herein, we adapt it here for the formation of acetyl phosphate from acetyl-CoA and inorganic phosphate (Fig 6). Prominent residues in this mechanism were S314, R315, and D321 (TP0094 numbering; the equivalent residues in MtPta were S309, R310, and D316). In the proposed mechanism, D321 abstracts a proton from bound inorganic phosphate, which then attacks bound acetyl-CoA, forming a tetrahedral intermediate that is stabilized by S314. R315 likely plays a role in binding and orienting the phosphate moiety throughout catalysis. In a final step, D321 protonates the sulfhydryl group of CoA, and the products dissociate.

The strict conservation of all three of these amino-acid residues (Fig 4) prompted us to mutate each of them individually and examine their respective enzyme activities. All three mutant proteins were similar in their hydrodynamic properties and thermal stabilities when compared to the wild-type enzyme (Table 4; S1 Fig). Also, they all had substantially curtailed enzyme activities compared to the wild-type enzyme, suggesting that the same mechanism of catalysis is operative in TP0094 and MtPta (Table 3).

**Table 4:** Protein denaturation monitored by CD.

Protein	*T*_m,app_ (K)
wild-type	321.03 [320.94, 320.12]^a^
S314A	320.6 [320.5, 320.7]
R315A	323.9 [323.8, 324.0]
D321A	317.27 [317.18, 317.36]

^a^Values in square brackets represent the 68.3% confidence interval from a single replicate.

## Conclusions

As part of the flavin-centric hypothesis of the physiology of *T*. *pallidum*, we have proposed that, in addition to glycolysis, an energy-conservation acetogenic pathway can also operate in the cytoplasm of this bacterium. This pathway is dependent on the catabolism of D-lactate, and it is capable of generating ATP in the *T*. *pallidum* cytoplasm via substrate-level phosphorylation and by contributing (via generated reducing equivalents) to the electrochemical gradient between the periplasm and the cytoplasm (Fig 1). Four enzymes are necessary for this proposed pathway to function efficiently: D-lactate dehydrogenase (DLD), pyruvate:flavodoxin oxidoreductase (PFOR), Pta, and Ack. We have previously validated the structure and activity of TP0037 as the DLD (TpDLD; [11]). In this work, we have focused on the proposed Pta activity of TP0094.

The structural and biochemical studies reported above were all compatible with TP0094’s role as the Pta of a the proposed acetogenic energy-conservation pathway (Fig 1). All structural information, including the X-ray crystal structure (Figs 2 & 3; Table 2) and *in vitro* biophysical assessment of the quaternary structure (Fig 5) was compatible with a dimeric assembly, like other known Pta enzymes [28–30]. Also, TP0094 catalyzed a reaction known to occur for other Ptas, i.e., the release of CoA from acetyl-CoA in the presence of inorganic phosphate; this activity was significantly diminished when putative active-site residues are altered (Table 4). From these facts, we infer that TP0094 can act as a *bona fide* Pta enzyme in *T*. *pallidum*, and we propose to refer to it as TpPta henceforth.

With the structural enzymology of TpDLD [11] and TpPta (this work) confirmed, one-half of the proposed flavin-dependent acetogenesis pathway in *T*. *pallidum* is verified. Hence, strong evidence for the existence of the pathway, and thus of the proposed flavin-dependent lifestyle of *T*. *pallidum*, currently exists. However, two remaining enzyme components of the pathway have yet to be characterized: PFOR (TP0939) and Ack (TP0476). Structural and biophysical studies are underway on these two proteins to provide complete confirmation of the pathway.

## Supporting information

S1 Fig Thermal denaturation curves for TP0094 and its mutants. Data points are represented by markers, and the lines are respective fits to the data using Eq 1 of the text. Respective colors and markers for each protein construct are alluded to in the inset legend.
(PDF)

S1 Table Primers used in this work. ^a^All sequences are given in the 5’-3’ orientation. ^b^Italicized lowercase letters indicate non-complementary nucleotides that were added for cloning purposes. ^c^Lowercase letters indicate nucleotides containing the mutation.
(PDF)

S2 Table The top 40 secondary-structure matching results from the search for structures similar to TP0094 (TpPta). ^a^% of secondary-structure elements matched; ^b^*Methanosarcina thermophila* phosphotransacetylase (Pta); ^c^*Porphyromonas gingivalis* Pta; ^d^*Bacillus subtilis* Pta; ^e^*Streptococcus pyrogenes* Pta; ^f^*Escherichia coli* EutD (no publication; 10.2210/pdb1VMI/pdb); ^g^*Staphylococcus aureus* Pta (no publication; 10.2210/pdb4E4R/pdb); ^h^*Enterococcus faecalis* branched-chain phosphotransacylase (no publication; 10.2210/pdb1YCO/pdb); ^i^*Listeria monocycogenes* Pta (no publications; 10.2210/pdb3U9E/pdb; 10.2210/pdb3UF6/pdb; 10.2210/pdb3TNG/pdb); ^j^*E*. *faecalis* PlsX; ^k^*Salmonella typhimurium* PdxA2 (no publication; 10.2210/pdb2HI1/pdb); ^l^*Burkholderia xenovorans* TphB; ^m^*E*. *coli* PdxA; ^n^*S*. *typhimurium* PdxA (no publication; 10.2210/pdb1R8K/pdb).
(PDF)

S3 Table Top 40 hits from a heuristic search (DALI) for comparable structures to TP0094 (TpPta). ^a^*M*. *thermophila* Pta; ^b^*P*. *gingivalis* Pta; ^c^*B*. *subtilis* Pta; ^d^*S*. *pyrogenes* Pta; ^e^*E*. *coli* Pta (no publication; 10.2210/pdb7T88/pdb); ^f^ *Escherichia coli* EutD (no publication; 10.2210/pdb1VMI/pdb); ^g^*Staphylococcus aureus* EutD (no publication; 10.2210/pdb4E4R/pdb); ^h^*Bdellovibrio bacteriovorus* MaeB PTA domain.
(PDF)

## References

[1] Schaudinn FN, Hoffmann E. Vorluafiger Bericht uber das Vorkommen von Spirochaeten in sphilitschen Krankheitsproduckten und bei Papillomen. Arb aus dem Kais Gesundheitsamte. 1905;22:527–34.

[2] Fraser CM, Norris SJ, Weinstock GM, White O, Sutton GG, Dodson R, et al. Complete genome sequence of *Treponema pallidum*, the syphilis spirochete. Science. 1998;281:375–88. 9665876 10.1126/science.281.5375.375

[3] Radolf JD, Deka RK, Anand A, Smajs D, Norgard M V., Yang XF. *Treponema pallidum*, the syphilis spirochete: making a living as a stealth pathogen. Nat Rev Microbiol. 2016;14:744–59. 27721440 10.1038/nrmicro.2016.141 PMC5106329

[4] Canale-Parola E. Motility and chemotaxis of spirochetes. Annu Rev Microbiol. 1978;32:69–99. doi: 10.1146/annurev.mi.32.100178.000441 360979

[5] Deka RK, Brautigam CA, Biddy BA, Liu WZ, Norgard MV. Evidence for an ABC-type riboflavin transporter system in pathogenic spirochetes. MBio. 2013;4:e00615–12. doi: 10.1128/mBio.00615-12 23404400 PMC3573665

[6] Duurkens RH, Tol MB, Geertsma ER, Permentier HP, Slotboom DJ. Flavin binding to the high affinity riboflavin transporter RibU. J Biol Chem. 2007;282:10380–6. doi: 10.1074/jbc.M608583200 17289680

[7] Burgess CM, Slotboom DJ, Geertsma ER, Duurkens RH, Poolman B, Van Sinderen D. The riboflavin transporter RibU in *Lactococcus lactis*: molecular characterization of gene expression and the transport mechanism. J Bacteriol. 2006;188:2752–60. 16585736 10.1128/JB.188.8.2752-2760.2006 PMC1446998

[8] Vogl C, Grill S, Schilling O, Stülke J, Mack M, Stolz J. Characterization of riboflavin (vitamin B2) transport proteins from *Bacillus subtilis* and *Corynebacterium glutamicum*. J Bacteriol. 2007;189:7367–75. 17693491 10.1128/JB.00590-07 PMC2168442

[9] Deka RK, Brautigam CA, Liu WZ, Tomchick DR, Norgard MV. The TP0796 lipoprotein of *Treponema pallidum* is a bimetal-dependent FAD pyrophosphatase with a potential role in flavin homeostasis. J Biol Chem. 2013 Apr 19;288:11106–21. 23447540 10.1074/jbc.M113.449975 PMC3630870

[10] Deka RK, Brautigam CA, Liu WZ, Tomchick DR, Norgard M V. Molecular insights into the enzymatic diversity of flavin-trafficking protein (Ftp; formerly ApbE) in flavoprotein biogenesis in the bacterial periplasm. Microbiologyopen. 2016;5:21–38. doi: 10.1002/mbo3.306 26626129 PMC4767422

[11] Deka RK, Liu WZ, Norgard M V., Brautigam CA. Biophysical and biochemical characterization of TP0037, a D-lactate dehydrogenase, supports an acetogenic energy conservation pathway in *Treponema pallidum*. MBio. 2020;11:e02249–20. 32963009 10.1128/mBio.02249-20 PMC7512555

[12] Deka RK, Brautigam CA, Yang XF, Blevins JS, Machius M, Tomchick DR, et al. The PnrA (Tp0319; TmpC) lipoprotein represents a new family of bacterial purine nucleoside receptor encoded within an ATP-binding cassette (ABC)-like operon in *Treponema pallidum*. J Biol Chem. 2006;281:8072–81. 16418175 10.1074/jbc.M511405200

[13] Klock HE, Koesema EJ, Knuth MW, Lesley SA. Combining the polymerase incomplete primer extension method for cloning and mutagenesis with microscreening to accelerate structural genomics efforts. Proteins Struct Funct Genet. 2008;71:982–94. doi: 10.1002/prot.21786 18004753

[14] Deka RK, Brautigam CA, Goldberg M, Schuck P, Tomchick DR, Norgard MV. Structural, bioinformatic, and *in vivo* analyses of two *Treponema pallidum* lipoproteins reveal a unique TRAP transporter. J Mol Biol. 2012;416:678–96. 22306465 10.1016/j.jmb.2012.01.015 PMC3289903

[15] Campos-Bermudez VA, Bologna FP, Andreo CS, Drincovich MF. Functional dissection of *Escherichia coli* phosphotransacetylase structural domains and analysis of key compounds involved in activity regulation. FEBS J. 2010;277:1957–66. 20236319 10.1111/j.1742-4658.2010.07617.x

[16] Zhao H, Ghirlando R, Piszczek G, Curth U, Brautigam CA, Schuck P. Recorded scan times can limit the accuracy of sedimentation coefficients in analytical ultracentrifugation. Anal Biochem. 2013;437(1):104–8. doi: 10.1016/j.ab.2013.02.011 23458356 PMC3676908

[17] Schuck P. Size distribution analysis of macromolecules by sedimentation velocity ultracentrifugation and Lamm equation modeling. Biophys J. 2000;78:1606–19. doi: 10.1016/S0006-3495(00)76713-0 10692345 PMC1300758

[18] Schuck P, Demeler B. Direct sedimentation analysis of interference optical data in analytical ultracentrifugation. Biophys J. 1999;76:2288–96. doi: 10.1016/S0006-3495(99)77384-4 10096923 PMC1300201

[19] Brautigam CA. Calculations and publication-quality illustrations for analytical ultracentrifugation data. Methods Enzymol. 2015;562:109–34. doi: 10.1016/bs.mie.2015.05.001 26412649

[20] Otwinowski Z, Minor W. Processing of X-ray diffraction data collected in oscillation mode. Methods Enzymol. 1997;276:307–26. doi: 10.1016/S0076-6879(97)76066-X 27754618

[21] French S, Wilson K. On the treatment of negative intensity observations. Acta Crystallogr A. 1978;34:517–25.

[22] McCoy AJ, Grosse-Kunstleve RW, Adams PD, Winn MD, Storoni LC, Read RJ. Phaser crystallographic software. J Appl Crystallogr. 2007 Aug 1;40(Pt 4):658–74. doi: 10.1107/S0021889807021206 19461840 PMC2483472

[23] Mirdita M, Schütze K, Moriwaki Y, Heo L, Ovchinnikov S, Steinegger M. ColabFold: making protein folding accessible to all. Nat Methods. 2022;19:679–82. doi: 10.1038/s41592-022-01488-1 35637307 PMC9184281

[24] Jumper J, Evans R, Pritzel A, Green T, Figurnov M, Ronneberger O, et al. Highly accurate protein structure prediction with AlphaFold. Nature. 2021;596:583–9. doi: 10.1038/s41586-021-03819-2 34265844 PMC8371605

[25] Adams PD, Afonine PV, Bunkóczi G, Chen VB, Davis IW, Echols N, et al. PHENIX: a comprehensive Python-based system for macromolecular structure determination. Acta Crystallogr Sect D Biol Crystallogr. 2010;66:213–21. 20124702 10.1107/S0907444909052925 PMC2815670

[26] Emsley P, Cowtan K. Coot: Model-building tools for molecular graphics. Acta Crystallogr Sect D Biol Crystallogr. 2004;60:2126–32. doi: 10.1107/S0907444904019158 15572765

[27] Xu QS, Shin DH, Pufan R, Yokota H, Kim R, Kim SH. Crystal structure of a phosphotransacetylase from *Streptococcus pyogenes*. Proteins Struct Funct Genet. 2004;55:479–81. 15048838 10.1002/prot.20039

[28] Iyer PP, Lawrence SH, Luther KB, Rajashankar KR, Yennawar HP, Ferry JG, et al. Crystal structure of phosphotransacetylase from the methanogenic archaeon *Methanosarcina thermophila*. Structure. 2004;12(4):559–67. 15062079 10.1016/j.str.2004.03.007

[29] Lawrence SH, Luther KB, Schindelin H, Ferry JG. Structural and functional studies suggest a catalytic mechanism for the phosphotransacetylase from *Methanosarcina thermophila*. J Bacteriol. 2006;188(3):1143–54. 16428418 10.1128/JB.188.3.1143-1154.2006 PMC1347337

[30] Yoshida Y, Sato M, Nonaka T, Hasegawa Y, Kezuka Y. Characterization of the phosphotransacetylase-acetate kinase pathway for ATP production in *Porphyromonas gingivalis*. J Oral Microbiol. 2019;11:1588086. 31007866 10.1080/20002297.2019.1588086 PMC6461089

[31] Krissinel E, Henrick K. Inference of Macromolecular Assemblies from Crystalline State. J Mol Biol. 2007;372:774–97. doi: 10.1016/j.jmb.2007.05.022 17681537

[32] Holm L, Rosenstrōm P. Dali server: conservation mapping in 3D. Nucleic Acids Res. 2010;38:W545–9. doi: 10.1093/nar/gkq366 20457744 PMC2896194

[33] Krissinel E, Henrick K. Secondary-structure matching (SSM), a new tool for fast protein structure alignment in three dimensions. Acta Crystallogr Sect D Biol Crystallogr. 2004;60:2256–68. doi: 10.1107/S0907444904026460 15572779

[34] Xu QS, Jancarik J, Lou Y, Kuznetsova K, Yakunin AF, Yokota H, et al. Crystal structures of a phosphotransacetylase from *Bacillus subtilis* and its complex with acetyl phosphate. J Struct Funct Genomics. 2005;6:269–79. 16283428 10.1007/s10969-005-9001-9

[35] Pei J, Kim BH, Grishin NV. PROMALS3D: a tool for multiple sequence and structure alignment. Nucleic Acids Res. 2008;36:2295–300. 18287115 10.1093/nar/gkn072 PMC2367709

[36] Robert X, Gouet P. Deciphering key features in protein structures with the new ENDscript server. Nucleic Acids Res. 2014;42:W320–4. doi: 10.1093/nar/gku316 24753421 PMC4086106

[37] Whiteley HR, Pelroy RA. Purification and properties of phosphotransacetylase from *Veillonella alcalescens*. J Biol Chem. 1972;247:1911–7. 5012766

[38] Harding CJ, Cadby IT, Moynihan PJ, Lovering AL. A rotary mechanism for allostery in bacterial hybrid malic enzymes. Nat Commun. 2021;12:1228. doi: 10.1038/s41467-021-21528-2 33623032 PMC7902834

