# Supplementary material for: Biophysical and biochemical studies support TP0094 as a phosphotransacetylase in an acetogenic energy-conservation pathway in Treponema pallidum
Source: PLoS One. 2023 May 18;18(5):e0283952. doi: 10.1371/journal.pone.0283952 (PMC10194888; doi:10.1371/journal.pone.0283952)
Supplement: S1 Fig — Data points are represented by markers, and the lines are respective fits to the data using Eq 1 of the text. Respective colors and markers for each protein construct are alluded to in the inset legend. (PDF) [file pone.0283952.s001.pdf]

Supplemental Figure

Figure S1.

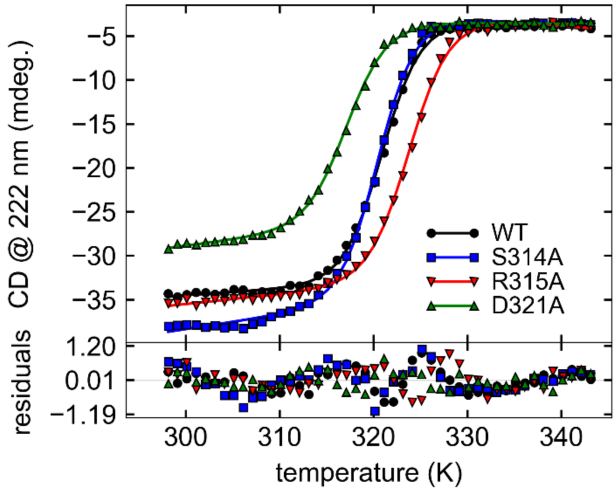

Fig S1. Thermal denaturation curves for TP0094 and its mutants. Data points are represented by markers, and the lines are respective fits to the data using Eq. 1 of the text. Respective colors and markers for each protein construct are alluded to in the inset legend.
