# Supplementary material for: Biophysical and biochemical studies support TP0094 as a phosphotransacetylase in an acetogenic energy-conservation pathway in Treponema pallidum
Source: PLoS One. 2023 May 18;18(5):e0283952. doi: 10.1371/journal.pone.0283952 (PMC10194888; doi:10.1371/journal.pone.0283952)
Supplement: S1 Table — aAll sequences are given in the 5’-3’ orientation. bItalicized lowercase letters indicate non-complementary nucleotides that were added for cloning purposes. cLowercase letters indicate nucleotides containing the mutation. (PDF) [file pone.0283952.s002.pdf]

Table S1. Primers used in this work.

**Primer identifier**	**Sequence**[a]
**PCR (cloning)**	
Tp0094pspeedP3	*ctgtacttccagggc*ATGACCTTCGTTGAATCAATGCAGCGG[b]
Tp0094pspeedP4	*AATTAAGTCGCGTTA*TTAGCGTCCATTCGATTGCACAAGTGT
**Site-directed Mutagenesis**	
Tp0094 S314A F	CCACTGTCTGATCTCgcgCGTGGGTGCTCGGTT[c]
Tp0094 S314A R	AACCGAGCACCCACGCGCGAGATCAGACAGTGG
Tp0094 R315A F	CTGTCTGATCTCTCGgctGGGTGCTCGGTTGAA
Tp0094 R315A R	TTCAACCGAGCACCCAGCCGAGAGATCAGACAG
Tp0094 D321A F	GGGTGCTCGGTTGAAgctATCGTCGCCGCTTGT
Tp0094 D321A R	ACAAGCGGCGACGATAGCTTCAACCGAGCACCC

[a]All sequences are given in the 5’-3’ orientation.
[b]Italicized lowercase letters indicate non-complementary nucleotides that were added for cloning purposes.
[c]Lowercase letters indicate nucleotides containing the mutation.
