# Supplementary material for: Biophysical and biochemical studies support TP0094 as a phosphotransacetylase in an acetogenic energy-conservation pathway in Treponema pallidum
Source: PLoS One. 2023 May 18;18(5):e0283952. doi: 10.1371/journal.pone.0283952 (PMC10194888; doi:10.1371/journal.pone.0283952)
Supplement: S2 Table — a% of secondary-structure elements matched; bMethanosarcina thermophila phosphotransacetylase (Pta); cPorphyromonas gingivalis Pta; dBacillus subtilis Pta; eStreptococcus pyrogenes Pta; fEscherichia coli EutD (no publication; 10.2210/pdb1VMI/pdb); gStaphylococcus aureus Pta (no publication; 10.2210/pdb4E4R/pdb); hEnterococcus faecalis branched-chain phosphotransacylase (no publication; 10.2210/pdb1YCO/pdb); iListeria monocycogenes Pta (no publications; 10.2210/pdb3U9E/pdb; 10.2210/pdb3UF6/pdb; 10.2210/pdb3TNG/pdb); jE. faecalis PlsX; kSalmonella typhimurium PdxA2 (no publication; 10.2210/pdb2HI1/pdb); lBurkholderia xenovorans TphB; mE. coli PdxA; nS. typhimurium PdxA (no publication; 10.2210/pdb1R8K/pdb). (PDF) [file pone.0283952.s003.pdf]

Table S2. The top 40 secondary-structure matching results from the search for structures similar to TP0094 (TpPta).

Hit #	Q-score	r.m.s.d. (Å)	# aligned C_α's	%SSE[a]	Accession:Chain	Identity
1	0.84	1.14	326	96	1qzt:C	MtPta[b]
2	0.81	1.31	327	96	1qzt:D	MtPta
3	0.81	1.28	325	100	2af3:C	MtPta
4	0.79	1.35	324	100	1qzt:A	MtPta
5	0.79	1.41	326	83	2af3:D	MtPta
6	0.78	1.4	324	100	2af4:C	MtPta
7	0.78	1.35	325	100	6iox:A	PgPta[c]
8	0.77	1.43	325	100	6iox:B	PgPta
9	0.75	1.47	324	100	6iow:A	PgPta
10	0.74	1.54	326	100	6iow:B	PgPta
11	0.73	1.61	323	96	2af4:D	MtPta
12	0.71	1.71	319	100	1td9:E	BsPta[d]
13	0.71	1.72	319	100	1td9:A	BsPta
14	0.71	1.71	319	100	1td9:B	BsPta
15	0.71	1.72	319	100	1xco:B	BsPta
16	0.71	1.72	319	100	1xco:E	BsPta
17	0.71	1.73	319	100	1td9:F	BsPta
18	0.71	1.74	319	100	1td9:C	BsPta
19	0.71	1.73	319	100	1xco:A	BsPta
20	0.71	1.75	319	100	1xco:C	BsPta
21	0.71	1.73	319	100	1td9:D	BsPta
22	0.7	1.76	319	100	1xco:F	BsPta
23	0.7	1.74	319	100	1xco:D	BsPta
24	0.67	1.84	318	96	1r5j:A	SpPta[e]
25	0.67	1.85	318	96	1r5j:B	SpPta
26	0.59	1.97	304	96	1vmi:A	EcEutD[f]
27	0.55	2.19	305	100	4e4r:A	SaPta[g]
28	0.54	2.39	313	96	1qzt:B	MtPta
29	0.41	2.59	251	79	1yco:B	EfBCPta[h]
30	0.36	2.92	257	79	3u9e:A	LmPta[i]
31	0.35	2.96	253	79	1yco:A	EfBCPta
32	0.35	2.96	257	79	3uf6:A	LmPta
33	0.35	2.97	259	79	3u9e:B	LmPta
34	0.35	2.95	256	79	3uf6:B	LmPta
35	0.35	2.94	255	79	3tng:A	LmPta
36	0.26	2.67	224	71	1u7n:A	EfPlsX[j]
37	0.24	2.97	225	75	2hi1:B	StPdxA2[k]
38	0.21	3.46	227	79	4aty:A	BxTphB[l]
39	0.21	2.99	216	79	1ps7:C	EcPdxA[m]
40	0.21	3.04	216	75	1r8k:A	StPdxA[n]

[a]% of secondary-structure elements matched; [b]*Methanosarcina thermophila* phosphotransacetylase (Pta) (1,2); [c]*Porphyromonas gingivalis* Pta (3); [d]*Bacillus subtilis* Pta (4); [e]*Streptococcus pyrogenes* Pta (5); [f]*Escherichia coli* EutD (no publication; 10.2210/pdb1VMI/pdb); [g]*Staphylococcus aureus* Pta (no publication; 10.2210/pdb4E4R/pdb); [h]*Enterococcus faecalis* branched-chain phosphotransacylase (no publication; 10.2210/pdb1YCO/pdb); [i]*Listeria monocycogenes* Pta (no publications; 10.2210/pdb3U9E/pdb; 10.2210/pdb3UF6/pdb; 10.2210/pdb3TNG/pdb); [j]*E. faecalis* PlsX (6); [k]*Salmonella typhimurium*

PdxA2 (no publication; 10.2210/pdb2HI1/pdb); [l]*Burkholderia xenovorans* TphB (7); [m]*E. coli* PdxA (8); [n]*S. typhimurium* PdxA (no publication; 10.2210/pdb1R8K/pdb)

References

1. Iyer PP, Lawrence SH, Luther KB, Rajashankar KR, Yennawar HP, Ferry JG, et al. Crystal structure of phosphotransacetylase from the methanogenic archaeon *Methanosarcina thermophila*. Structure. 2004;12(4):559–67.

2. Lawrence SH, Luther KB, Schindelin H, Ferry JG. Structural and functional studies suggest a catalytic mechanism for the phosphotransacetylase from *Methanosarcina thermophila*. J Bacteriol. 2006;188(3):1143–54.

3. Yoshida Y, Sato M, Nonaka T, Hasegawa Y, Kezuka Y. Characterization of the phosphotransacetylase-acetate kinase pathway for ATP production in *Porphyromonas gingivalis*. J Oral Microbiol. 2019;11:1588086.

4. Xu QS, Jancarik J, Lou Y, Kuznetsova K, Yakunin AF, Yokota H, et al. Crystal structures of a phosphotransacetylase from *Bacillus subtilis* and its complex with acetyl phosphate. J Struct Funct Genomics. 2005;6:269–79.

5. Xu QS, Shin DH, Pufan R, Yokota H, Kim R, Kim SH. Crystal structure of a phosphotransacetylase from *Streptococcus pyogenes*. Proteins Struct Funct Genet. 2004;55:479–81.

6. Kim Y, Li H, Binkowski TA, Holzle D, Joachimiak A. Crystal structure of fatty acid/phospholipid synthesis protein PlsX from *Enterococcus faecalis*. J Struct Funct Genomics. 2009;10:157–63.

7. Bains J, Wulff JE, Boulanger MJ. Investigating terephthalate biodegradation: structural characterization of a putative decarboxylating cis-dihydrodiol dehydrogenase. J Mol Biol. 2012;423:284–93.

8. Sivaraman J, Li Y, Banks J, Cane DE, Matte A, Cygler M. Crystal structure of Escherichia coli PdxA, an enzyme involved in the pyridoxal phosphate biosynthesis pathway*. J Biol Chem. 2003;278:43682–90.

9. Harding CJ, Cadby IT, Moynihan PJ, Lovering AL. A rotary mechanism for allostery in bacterial hybrid malic enzymes. Nat Commun. 2021;12:1228.
