# Supplementary material for: Biophysical and biochemical studies support TP0094 as a phosphotransacetylase in an acetogenic energy-conservation pathway in Treponema pallidum
Source: PLoS One. 2023 May 18;18(5):e0283952. doi: 10.1371/journal.pone.0283952 (PMC10194888; doi:10.1371/journal.pone.0283952)
Supplement: S3 Table — aM. thermophila Pta; bP. gingivalis Pta; cB. subtilis Pta; dS. pyrogenes Pta; eE. coli Pta (no publication; 10.2210/pdb7T88/pdb); f Escherichia coli EutD (no publication; 10.2210/pdb1VMI/pdb); gStaphylococcus aureus EutD (no publication; 10.2210/pdb4E4R/pdb); hBdellovibrio bacteriovorus MaeB PTA domain. (PDF) [file pone.0283952.s004.pdf]

Table S3. Top 40 hits from a heuristic search (DALI) for comparable structures to TP0094 (TpPta).

Hit #	PDB Code-Chain	Z-score	r.ms.d (Å)	# aligned C_α's	Identity
1	1qzt-C	46.8	1.2	327	MtPta[a]
2	6iox-A	46.7	1.4	329	PgPta[b]
3	6iox-B	46.6	1.5	329	PgPta
4	2af3-C	46.5	1.3	327	MtPta
5	6iow-A	46.2	1.5	329	PgPta
6	1qzt-D	46.2	1.3	327	MtPta
7	2af4-C	45.8	1.5	327	MtPta
8	1qzt-A	45.7	1.4	327	MtPta
9	6iow-B	45.5	1.6	328	PgPta
10	2af4-D	45.4	1.6	327	MtPta
11	2af3-D	44.9	1.4	327	MtPta
12	1xco-A	43.1	1.8	321	BsPta[c]
13	1td9-E	43.1	1.8	321	BsPta
14	1td9-A	43.1	1.8	321	BsPta
15	1td9-B	43.1	1.8	321	BsPta
16	1xco-E	43.1	1.8	321	BsPta
17	1td9-C	43.1	1.8	321	BsPta
18	1xco-C	43.1	1.8	321	BsPta
19	1qzt-B	43	2.7	327	MtPta
20	1xco-B	43	1.8	321	BsPta
21	1td9-F	43	1.8	321	BsPta
22	1xco-F	42.9	1.8	321	BsPta
23	1td9-D	42.9	1.8	321	BsPta
24	1xco-D	42.9	1.8	321	BsPta
25	1r5j-B	42.6	2	323	SpPta[d]
26	1r5j-A	42.6	1.9	323	SpPta
27	7t88-A	42.3	2.2	320	EcPta[e]
28	1vmi-A	40.7	2.3	321	EcEutD[f]
29	4e4r-A	40.6	2.3	318	SaEutD[g]
30	6zn9-K	39.2	2.2	319	BbMaeB[h]
31	6znk-B	39.2	2.1	319	BbMaeB
32	6zn9-G	39.1	2.1	319	BbMaeB
33	6znk-E	39.1	2.1	319	BbMaeB
34	6znk-J	39.1	2.2	319	BbMaeB
35	6zn9-E	39	2.2	319	BbMaeB
36	6zn9-H	39	2.2	319	BbMaeB
37	6znk-C	38.9	2.1	319	BbMaeB
38	6znk-D	38.8	2.1	319	BbMaeB
39	6znk-A	38.8	2.2	319	BbMaeB
40	6znk-H	38.8	2.3	319	BbMaeB

[a]*M. thermophila* Pta (1,2); [b]*P. gingivalis* Pta (3); [c]*B. subtilis* Pta (4); [d]*S. pyrogenes* Pta (5); [e]*E. coli* Pta (no publication; 10.2210/pdb7T88/pdb); [f] *Escherichia coli* EutD (no publication; 10.2210/pdb1VMI/pdb); [g]*Staphylococcus aureus* EutD (no publication; 10.2210/pdb4E4R/pdb); [h]*Bdellovibrio bacteriovorus* MaeB PTA domain (9)

References

1. Iyer PP, Lawrence SH, Luther KB, Rajashankar KR, Yennawar HP, Ferry JG, et al. Crystal structure of phosphotransacetylase from the methanogenic archaeon *Methanosarcina thermophila*. Structure. 2004;12(4):559–67.

2. Lawrence SH, Luther KB, Schindelin H, Ferry JG. Structural and functional studies suggest a catalytic mechanism for the phosphotransacetylase from *Methanosarcina thermophila*. J Bacteriol. 2006;188(3):1143–54.

3. Yoshida Y, Sato M, Nonaka T, Hasegawa Y, Kezuka Y. Characterization of the phosphotransacetylase-acetate kinase pathway for ATP production in *Porphyromonas gingivalis*. J Oral Microbiol. 2019;11:1588086.

4. Xu QS, Jancarik J, Lou Y, Kuznetsova K, Yakunin AF, Yokota H, et al. Crystal structures of a phosphotransacetylase from *Bacillus subtilis* and its complex with acetyl phosphate. J Struct Funct Genomics. 2005;6:269–79.

5. Xu QS, Shin DH, Pufan R, Yokota H, Kim R, Kim SH. Crystal structure of a phosphotransacetylase from *Streptococcus pyogenes*. Proteins Struct Funct Genet. 2004;55:479–81.

6. Kim Y, Li H, Binkowski TA, Holzle D, Joachimiak A. Crystal structure of fatty acid/phospholipid synthesis protein PlsX from *Enterococcus faecalis*. J Struct Funct Genomics. 2009;10:157–63.

7. Bains J, Wulff JE, Boulanger MJ. Investigating terephthalate biodegradation: structural characterization of a putative decarboxylating cis-dihydrodiol dehydrogenase. J Mol Biol. 2012;423:284–93.

8. Sivaraman J, Li Y, Banks J, Cane DE, Matte A, Cygler M. Crystal structure of Escherichia coli PdxA, an enzyme involved in the pyridoxal phosphate biosynthesis pathway*. J Biol Chem. 2003;278:43682–90.

9. Harding CJ, Cadby IT, Moynihan PJ, Lovering AL. A rotary mechanism for allostery in bacterial hybrid malic enzymes. Nat Commun. 2021;12:1228.
